# *FAT2* mutation is associated with better prognosis and responsiveness to immunotherapy in uterine corpus endometrial carcinoma

**DOI:** 10.1002/cam4.5119

**Published:** 2022-08-07

**Authors:** Zhe Wang, Linan Xing, Yujie Huang, Peilin Han

**Affiliations:** ^1^ Department of Gynecology, The First Affiliated Hospital Zhejiang University School of Medicine Hangzhou People's Republic of China

**Keywords:** *FAT2* mutation, immunotherapy, prognosis, uterine corpus endometrial carcinoma

## Abstract

**Background:**

Uterine corpus endometrial carcinoma (UCEC) ranks sixth among malignant tumors in women and the mortality is still rising. *FAT2* gene has been considered to be related to the survival and prognosis of some certain diseases in previous studies, but the *FAT2* mutation status in UCEC and its prognostic value has been rarely studied. Hence, the purpose of our study was to explore the role of *FAT2* mutations for predicting prognosis and responsiveness to immunotherapy in patients with UCEC.

**Methods:**

UCEC samples from the Cancer Genome Atlas database were analyzed. We evaluated the impact of *FAT2* gene mutation status and clinicopathological characteristics on the prognosis of UCEC patients and used univariate and multivariate Cox analysis risk scores to independently predict patient overall survival (OS). Tumor mutation burden (TMB) values of the *FAT2* mutant and non‐mutant groups were computed by Wilcoxon rank sum test. The correlation of *FAT2* mutation and half maximal inhibitory concentration (IC50) values of various anticancer drugs was analyzed. Gene Ontology data and Gene Set Enrichment Analysis (GSEA) were employed to examine the differential expression of genes between the two groups. Finally, a single‐sample GSEA arithmetic was utilized to measure the abundance of tumor‐infiltrating immune cells in UCEC patients.

**Results:**

*FAT2* mutations suggested better OS (*p* < 0.001) and disease‐free survival (DFS) (*p* = 0.007) in UCEC. The IC50 values of 18 anticancer drugs were upregulated in *FAT2* mutation patients (*p* < 0.05). The TMB and microsatellite instability values of patients with *FAT2* mutations were significantly higher (*p* < 0.001). Next, the Kyoto Encyclopedia of Genes and Genomes functional analysis and GSEA revealed the potential mechanism of *FAT2* mutation on the tumorigenesis and progression of UCEC. In addition, in reference to the UCEC microenvironment, the infiltration levels of activated CD4/CD8 T cells (*p* < 0.001/*p* = 0.001) and plasmacytoid dendritic cells (*p* = 0.006) were upregulated in the non‐FAT2 mutation group, and Type 2 T helper cells (*p* = 0.001) were downregulated in the *FAT2* mutation group.

**Conclusions:**

UCEC patients with *FAT2* mutations have better prognosis and are more likely to respond to immunotherapy. *FAT2* mutation may be a valuable predictor for prognosis and responsiveness to immunotherapy in UCEC patients.

## Graphical abstract

UCEC patients with *FAT2* mutations have better prognosis. *FAT2* mutation may be an independent prognostic predictor for UCEC.

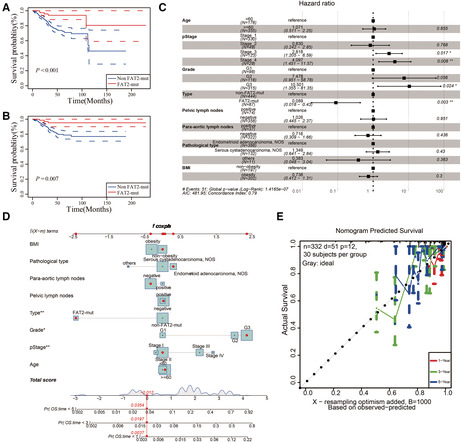

## 1 INTRODUCTION

Uterine corpus endometrial carcinoma (UCEC) ranks sixth among malignant tumor in women.^1^ In 2020, 417,367 women were diagnosed with UCEC globally, and the disease burden was highest in Western Europe and North America.^1^ According to a pooled analysis of epidemiological research in 2016, UCEC‐related mortality increased year by year since 1971 to 2014 at an average rate of 1.9%.^2^ This phenomenon indicates more effective strategies to diagnose and treat this disease are needed. For patients in early stages, the primary treatment is radical surgery which makes the 5‐year overall survival (OS) rate surpass 90%.^3^ Depending on the risk factors of disease, adjuvant radiation may be used alone or in combination with chemotherapy to reduce the chance of recurrence.^4^ Multiple prospective research has attempted to identify women in the early stage of disease with high risk for recurrence and to develop effective adjuvant therapeutic strategies. However, there has been no high‐level evidence supporting that adjuvant radiation and/or chemotherapy could improve OS so far. Clinical outcomes are substantially worse for patients with recurrent, advanced, or clinically aggressive tumors.^5^ There are few options for metastatic disease other than chemotherapy and endocrine therapy.^6^ Recently, there have been promising results from immune checkpoint inhibitor (ICI) trials for gynecological cancers, particularly UCEC and cervical cancers. Gynecological cancers represent a heterogeneous group of tumors, and their response to ICI can be predicted by using several biomarkers. However, the optimal biomarkers for specific types of cancer including UCEC have not been fully identified.^7^, ^8^ Therefore, it is pressing to find new potential markers to guide precise treatment to increase the ratio of UCEC patients who respond to immunotherapy.

*FAT2* belongs to the human *FAT* gene family. It was determined as the second human homolog of the *Drosophila FAT* gene with a complete coding sequence.^9^, ^10^ *FAT2* is located on human chromosome 5q33.1 and enables transcription of a 14.5‐kbp mRNA which involves an open reading frame (ORF) of 4349 amino acids.^9^ *FAT2* gene is widely expressed in human normal tissues such as brain tissue and tumor tissues such as esophageal carcinoma, gastric cancer, and ovarian cancer.^10^ *FAT2* has been detected mutated in malignant mesothelioma, esophageal squamous cell carcinoma, and colorectal carcinoma.^11^ In previous studies, it was found that the mutation and expression of *FAT2* is closely linked to the survival and prognosis of these certain diseases. The mutation and expression of *FAT2* could affect the survival and prognosis of gastric cancer patients,^11^, ^12^ for which *FAT2* mutation was associated with better patient prognosis. In a study investigating acute myeloid leukemia, *FAT2* was employed as an independent predictor for prognosis.^13^ Highly expressed *FAT2* in breast cancer and lung squamous cell carcinoma had poor patient outcomes.^14^ In esophageal squamous cell adenocarcinoma and carcinoma, patients with *FAT2* mutations had significantly worse OS.^15^ In colorectal cancer, mutation of *FAT2* in somatic cells may promote metastasis.^16^ Moreover, *FAT2* has been shown to predict responsiveness to immunotherapy in addition to disease survival and prognosis. In lung adenocarcinoma (LUAD), *FAT2* mutations could predict prognosis independently, which was correlated with higher tumor mutation burden (TMB) and tumor‐infiltrating immune cells (TICs). In NSCLC cohort treated with ICI, *FAT2* mutations may predict better clinical beneficial response and longer progression‐free survival.^17^ Based on the above information, we infer that *FAT2* may get involved in the tumorigenesis, progression, and treatment of UCEC.

In the present study, gene mutations in patients with UCEC were analyzed by using data from The Cancer Genome Atlas, and then, the impacts of *FAT2* gene mutations and clinicopathological features on the prognosis of UCEC were evaluated. Then, TMB and microsatellite instability (MSI) values of these patients with or without *FAT2* mutation were computed and compared. To resolve the potential mechanism of *FAT2* mutation in UCEC, we conducted KEGG functional analysis and gene set enrichment analysis (GSEA). In the end, the association of *FAT2* mutation status with immune cell infiltration was investigated. To sum up, our study may identify a potential marker to predict the prognosis of UCEC and responsiveness to immunotherapy.

## 2 MATERIALS AND METHODS

### 2.1 Data download and processing

The somatic mutation data of UCEC patients were acquired from The Cancer Genome Atlas (TCGA) official website (https://portal.gdc.cancer.gov/). We employed VarScan software to process the above data and R's maftools package to visualize somatic mutations.^18^ After downloading the gene expression data (FPKM value) of the patient's RNA sequencing, we converted FPKM value into TPM value and split it into mRNA and lncRNA's expression. In addition, we logged in UCSC Xena website (http://xena.ucsc.edu/) to download the clinicopathological characteristics, such as age, grade, TNM stage, and MSI value, and clinical outcome of the corresponding UCEC patients.

R's TCGAbiolinks package was employed to obtain the patient's Masked Copy Number Segment data. GenePattern5 was employed to conduct Genomic Identification of Significant Targets in Cancer (GISTIC) 2.0 analysis on the downloaded copy number variation (CNV) fragments.^19^ In the analysis procedure, several parameters such as the confidence level and the X chromosome (reserved before the analysis) were excluded, and we applied the default settings for the GISTIC 2.0 analysis. Finally, R's maftools package was used to visualize the result of GISTIC 2.0 analysis.^18^ We predicted the probability of each sample's response to immunotherapy based on the tracking of indels by decomposition (TIDE) algorithm (http://tide.dfci.harvard.edu).^20^

### 2.2 Determination of frequently mutated genes

UCEC samples were divided into two groups according to *FAT2* mutation status.^21^ R's DESeq2 package was analyzed for differentially expressed genes (DEGs) between groups, where log fold change (logFC) > 1.0 and *p* value <0.05 were defined as the thresholds of differential genes. Heatmap and volcano were used to visualize the results.

### 2.3 Clinical prediction model of FAT2 gene mutation's validation

We evaluated *FAT2* gene mutation status and clinicopathological characteristics on the prognosis of UCEC patients from the TCGA dataset and then applied univariate and multivariate Cox analysis risk scores to predict patients' OS. Next, we constructed a nomogram for survival prediction in patients from the TCGA dataset. Harrell's consistency index (C‐index) was employed for quantification of the discrimination performance. Meanwhile, we generated a calibration curve to testify the predicted value of the nomogram through comparing with the observed actual survival rate. In addition, ROC analyses were used to evaluate the individual prognostic factors and verify the accuracies of the nomogram.

### 2.4 Anticancer drug sensitivity analysis

The Genomics of Drug Sensitivity in Cancer (GDSC) database (https://www.cancerrxgene.org/) was accessed via R's pRRophetic package to acquire data of cell line gene mutation and IC50 values of various anticancer drugs.^22^ We analyzed the relationship between *FAT2* gene mutations and sensitivity to various anticancer drugs.

### 2.5 Correlation between FAT2 mutation and TMB

The total number of somatic mutations detected in each sample was defined as the sample's TMB. We computed the TMB value of each tumor sample and then compared the overall difference in TMB levels between the *FAT2* mutant and non‐mutant groups by Wilcoxon rank sum test.

### 2.6 Functional enrichment analysis and gene set enrichment analysis

GO DEG analysis results in this study are listed in Table S1. ClusterProfiler R software package was employed to run GO annotation analysis and KEGG pathway enrichment analysis for DEGs (Table S2).^23^ A cutoff value of FDR < 0.05 was defined as statistically significant.

For GSEA analysis of the dataset of TCGA‐UCEC patients' gene expression, the “c2.cp.kegg.v6.2.‐symbols” gene set was downloaded from the MSigDB database, and the adjusted *p* value <0.05 was defined as statistically significant (Table S3).^24^, ^25^

### 2.7 Construction of protein–protein interaction network and sifting of core genes (Hub genes)

The online Search Tool for the Retrieval of Interacting Genes/Proteins (STRING) was employed to predict and then construct a protein–protein interaction (PPI) network of selected genes.^26^ We chose genes with a score higher than 0.4 to build a visualized network model by Cytoscape (v3.7.2) in the STRING database.^27^

### 2.8 Construction of competing endogenous RNA network

We downloaded information about miRNA‐mRNA interactions from the miRTarBase database. Based on the core mRNA obtained by PPI interaction analysis, we predicted the regulated miRNA and related lncRNA through the miRTarBase database. R's ggalluvial package was employed to draw Sankey diagrams and visualize the results of competing endogenous RNA (ceRNA) analysis.

### 2.9 Evaluation of immune cell infiltration

Single‐sample GSEA (ssGSEA) algorithm was employed to quantify the relative abundance of TICs in UCEC patients. This algorithm contained in Bindea et al.'s research was used to obtain 28 gene sets for marking different TIC types.^28^ There were various human immune cell subtypes in the gene set, including CD8 T lymphocytes, dendritic lymphocytes, macrophages, etc. We calculated the enrichment score via the ssGSEA analysis in R's gsva package to express the infiltration level of the certain immune cell type.

### 2.10 Statistical analysis

The whole data processing and analysis in this study were implemented via R software (version 4.0.2). Student's *t*‐test was employed for comparison of continuous variables. Mann–Whitney *U* test (the Wilcoxon rank sum test) was employed to analyze the difference between non‐normally distributed variables. Chi‐square test or Fisher's exact test was employed for comparison and analysis of statistical significance between categorical variables. We computed the correlation coefficient among involved genes through Pearson correlation analysis. The survival package of R was employed for survival analysis. We drew the difference in survival using a Kaplan–Meier survival curve. Log‐rank test was employed to measure the significance of the difference in survival term between the two groups of patients. Univariate and multivariate Cox analyses were applied to identify independent prognostic factors. R's pROC package was employed to measure the accuracy of the risk score to make estimation of the prognosis by depicting the receiver operating characteristic (ROC) curve and computing the area under the curve (AUC).^29^ In this study, *p* value <0.05 was defined as statistically significant, and all statistical *p* values were two sided.

## 3 RESULTS

### 3.1 Overall mutation level of UCEC patients with or without FAT2 mutation

**FIGURE 1:**
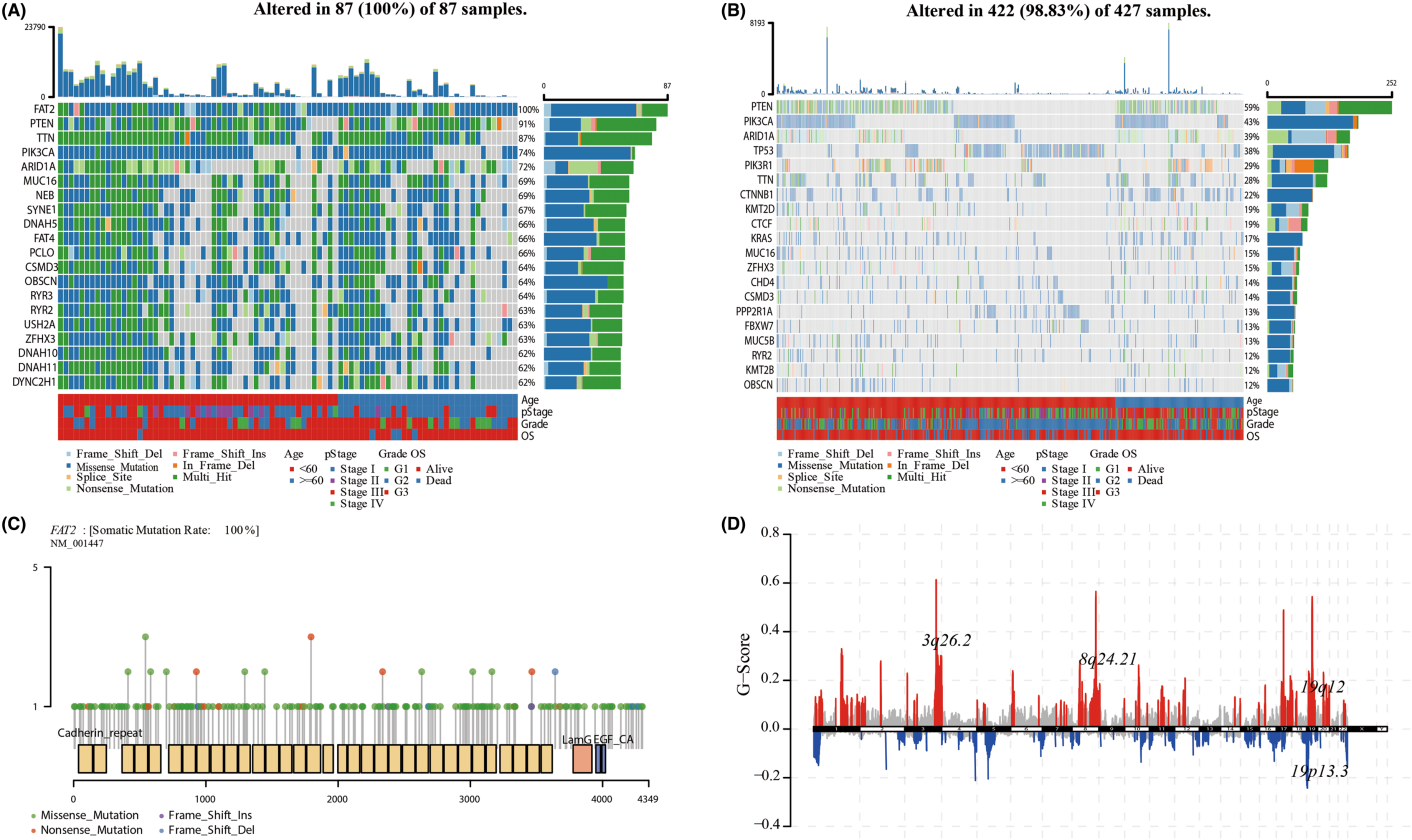
Landscapes of *FAT2* mutant or non‐mutant group and clinical correlation analysis in uterine corpus endometrial carcinoma (UCEC) patients. (A, B) In The Cancer Genome Atlas (TCGA)‐UCEC patient cohort, the first 20 significantly mutated genes in *FAT2* mutant patients and non‐*FAT2* mutant patients, of which the left side was the *FAT2* gene mutant group, and the right side was the non‐*FAT2* mutant group. Samples were sorted according to somatic non‐synonymous mutation burden, and genes were sorted according to mutation frequency. The section above the legend showed the sudden change load, and the age, stage, grade, and OS status were marked in the figure in that order; (C) The amino acid variation distribution map of the *FAT2* protein in the TCGA‐UCEC dataset, which was mainly in the form of missense mutations; (D) Maftools visualized the overall horizontal copy number variation results of GISTIC 2.0 based on the TCGA‐UCEC cohort.

The mutation data of 531 patients in the UCEC dataset from TCGA database were downloaded. Patients were divided into a *FAT2* mutation group and a non‐*FAT2* mutation group (clinical data are presented in Table 1). The top 20 frequently mutated genes are shown in Figure 1A,B. The three genes (*PTEN*, *PIK3CA*, and *ARIDI1)* rank in the top five frequently mutated genes of UCEC patients regardless of *FAT2* mutation status. We found that the frequency of *TP53* mutation was significantly lower in the *FAT2* mutation group. Changes of the amino acid in the *FAT2* protein indicated that the most common mutation form of the *FAT2* protein was concentrated in the missense mutation form (Figure 1C). Figure 1D shows that the CNV levels of many genes had changed significantly by comparing the two groups.

**TABLE 1:** The clinical and pathological characteristics of UCEC patients based on TCGA data

Characteristic	Non‐FAT2 mutation	FAT2 mutation
*n*	444	87
Age, *n* (%)		
<60	123 (23.2%)	53 (10%)
≥60	321 (60.5%)	34 (6.4%)
Stage, *n* (%)		
Stage I	278 (52.4%)	52 (9.8%)
Stage II	39 (7.3%)	10 (1.9%)
Stage III	101 (19%)	22 (4.1%)
Stage IV	26 (4.9%)	3 (0.6%)
Grade, *n* (%)		
G1	85 (16%)	13 (2.4%)
G2	101 (19%)	17 (3.2%)
G3	258 (48.6%)	57 (10.7%)
Pelvic lymph nodes, *n* (%)		
Negative	297 (68.8%)	61 (14.1%)
Positive	62 (14.4%)	12 (2.8%)
Para‐aortic lymph nodes, *n* (%)		
Negative	271 (75.3%)	52 (14.4%)
Positive	30 (8.3%)	7 (1.9%)
Pathological type, *n* (%)		
Endometrioid adenocarcinoma, NOS	313 (58.9%)	75 (14.1%)
Others	10 (1.9%)	1 (0.2%)
Serous cystadenocarcinoma, NOS	121 (22.8%)	11 (2.1%)
BMI, *n* (%)		
Non‐obesity (<30 kg/m^2^)	158 (31.7%)	39 (7.8%)
Obesity (≥30 kg/m^2^)	261 (52.3%)	41 (8.2%)

### 3.2 Relationship between FAT2 gene mutation and prognosis

**FIGURE 2:**
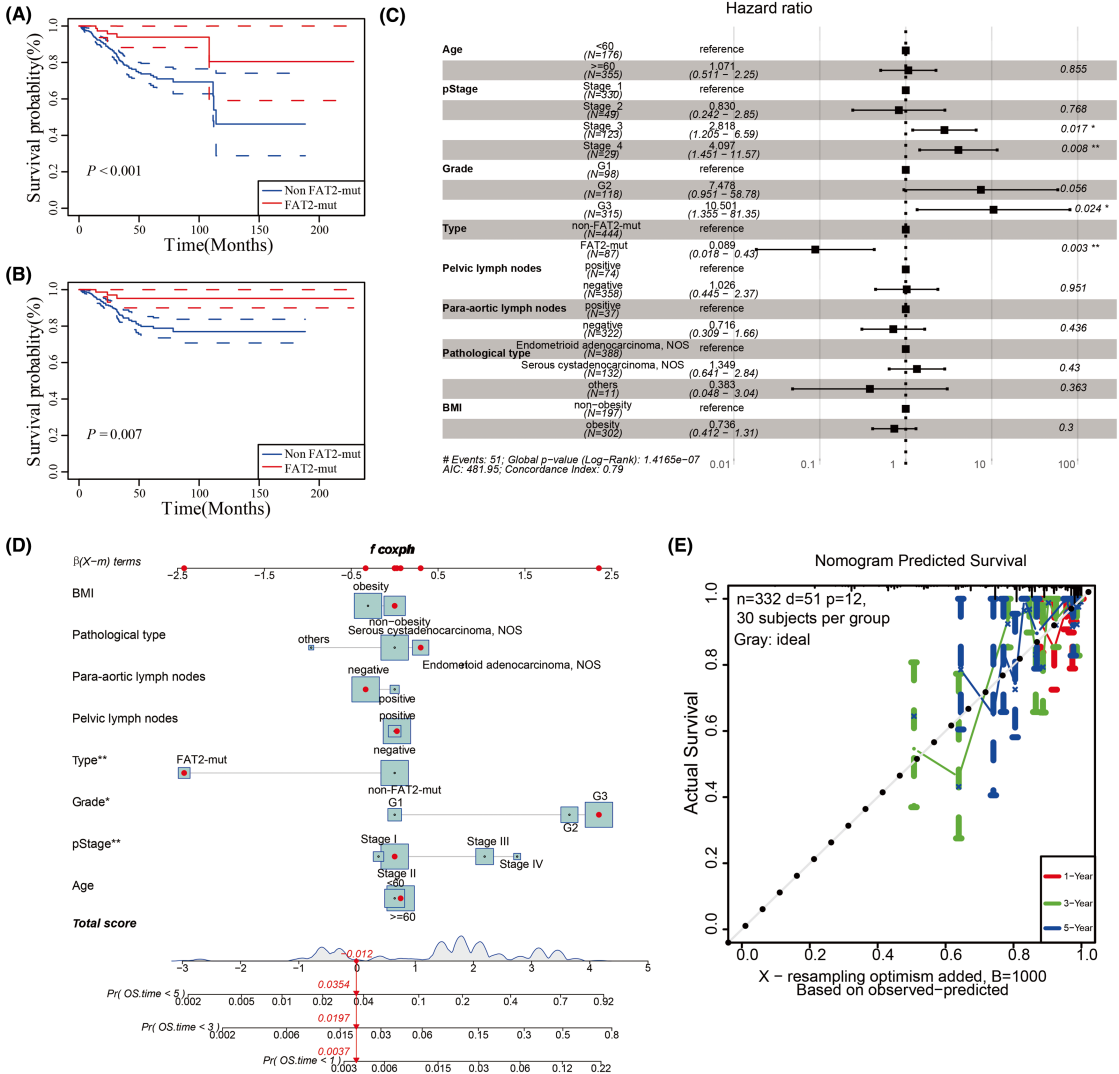
*FAT2* gene mutation's effect on clinicopathological characteristics in the TCGA‐UCEC dataset. (A, B) Survival analysis results showed that overall survival (OS, Log‐rank *p* < 0.001) and disease‐free survival (DFS, Log‐rank *p* = 0.007) of patients with *FAT2* gene mutations were better than patients without *FAT2* gene mutations in the TCGA‐UCEC dataset; (C) Multivariate Cox analysis showed that *FAT2* gene mutation status, stage, and grade were independent protective prognostic factors in UCEC patients; (D) The nomogram of OS was constructed by combining *FAT2* gene mutation and clinicopathological characteristics; (E) Calibration curve of the *FAT2* gene mutation nomogram. The abscissa was the survival situation predicted by the nomogram, and the ordinate was the survival situation actually observed. Repeated 1000 times, the curve showed that the prognosis of patients at 1, 3, and 5 years was well predicted by the nomogram.

We validated the prognostic value of *FAT2* mutations for UCEC patients from the TCGA‐UCEC dataset: *FAT2* mutations suggested better OS (Log‐rank *p* < 0.001; Figure 2A) and disease‐free survival (DFS; Log‐rank *p* = 0.007; Figure 2B). For further investigating the impact of *FAT2* mutations, we summarized the clinicopathological characteristics (Table 1), then univariate and multivariate Cox regression analysis showed that *FAT2* mutation status was an independent protective factor in UCEC (Figure 2C) (Table S4). We incorporated the *FAT2* mutation and clinicopathological characteristics into the model (Figure 2D) and built a nomogram for predicting the OS of UCEC patients. C‐Index was employed to compute the discriminative ability of the nomogram and revealed a certain discrimination degree of 0.778 (95% confidence interval: 0.729–0.827). In the calibration plot, bias‐corrected line and ideal curve were very close, which hinting favorable agreement between the predicted value and observed value (Figure 2E). Moreover, the time‐dependent ROC of *FAT2* mutation for OS and DFS in 1, 3, and 5 years was assessed by ROC package; *FAT2* mutation showed a better 1‐year, 3‐year, and 5‐year OS (AUC = 0.626, 0.654, and 0.797), and 1‐year, 3‐year, and 5‐year DFS (AUC = 0.605, 0.624, and 0.665) (Figure S1).

### 3.3 Anticancer drug sensitivity in patients with FAT2 gene mutation

**FIGURE 3:**
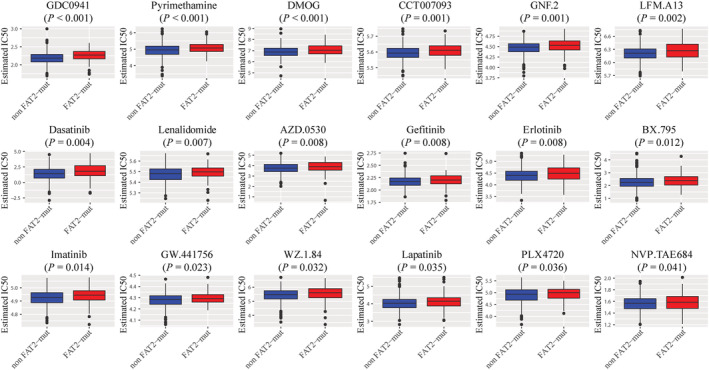
Based on the Genomics of Drug Sensitivity in Cancer (GDSC) database's analysis, the sensitivity of *FAT2* mutations to different chemotherapy drugs and small molecule anticancer drugs.

To assess the impact of *FAT2* mutations on the sensitivity of patients to the drug in UCEC, data of cell line gene mutation and IC50 values of various anticancer drugs were acquired from the GDSC database. Based on responsiveness of cell lines to 138 different anticancer drugs, we predicted the drug IC50 values in TCGA‐UCEC patients. The IC50 values of 18 anticancer drugs were significantly higher in *FAT2* mutation patients (*p* < 0.05; Figure 3), especially GDC0941, pyrimethamine, and DMOG (Figure 3).

### 3.4 Relationship between FAT2 gene mutation and TBM, MSI, and CNV score

**FIGURE 4:**
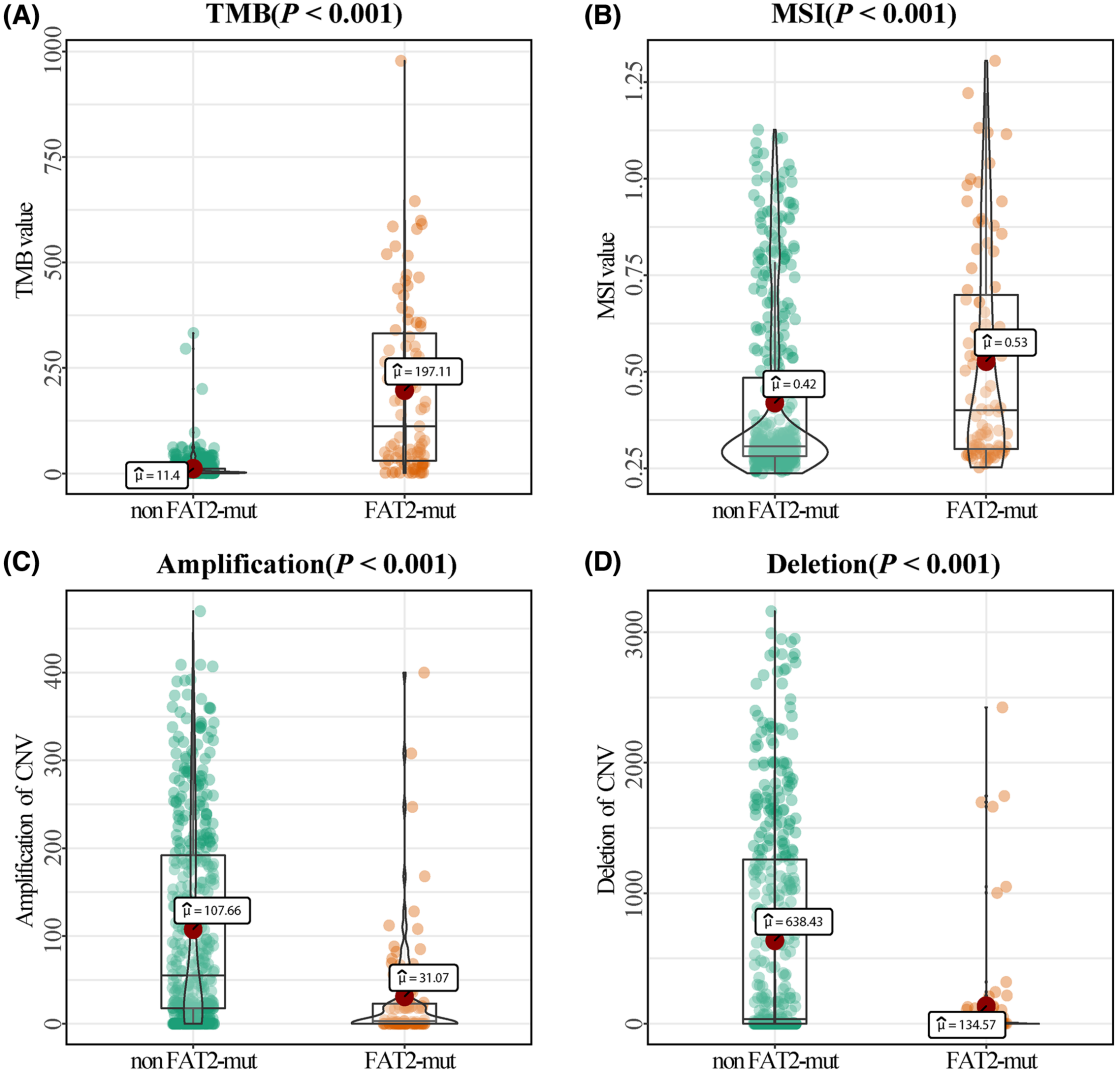
Tumor mutation burden (TMB) and microsatellite instability (MSI) of *FAT2* gene mutations' analysis in UCEC patients. (A) Compared with non‐mutated patients, the TMB level of *FAT2* mutant patients was significantly higher (*p* < 0.001); (B) *FAT2* mutation patients' MSI value was significantly increased (*p* < 0.001); (C) *FAT2* mutation patients' CNV amplification levels were significantly reduced (*p* < 0.001); (D) Patients with *FAT2* mutations had a reduced level of copy number variation deletion (*p* < 0.001).

We further analyzed the TMB, MSI, and CNV scores between the *FAT2* mutation group and the non‐*FAT2* mutation group. The values of TMB and MSI (*p* < 0.001; Figure 4A,B) of *FAT2* mutation patients were significantly higher. Moreover, the copy number increase or deletion level of CNV in *FAT2* mutation patients was significantly lower (*p* < 0.001; Figure 4C,D).

### 3.5 Analysis of differential expression in patients with FAT2 gene mutation

**FIGURE 5:**
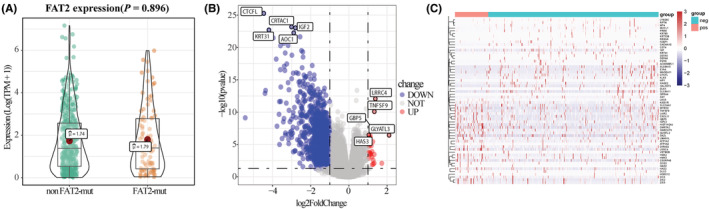
Differential expression analysis based on *FAT2* mutation. (A) Compared with that of non‐mutated patients, there was no significant difference in *FAT2* expression levels in *FAT2* mutated patients (*p* = 0.896); (B) Volcano map showed differentially expressed gene (DEG) expression between the *FAT2* gene mutation and non‐*FAT2* mutation groups. (C) The heatmap showed the overall expression characteristics of DEGs between the *FAT2* gene mutation and non‐mutation groups, in which the darker red represented the higher expression level, and the darker blue represented the lower expression level.

The differential expression analysis of TCGA‐UCEC dataset showed that there was no significant difference in *FAT2* expression levels between the two groups (Figure 5A). Between the two groups of patients, the expressions of 29 genes were upregulated significantly, yet the expressions of 1097 genes were downregulated significantly (Figure 5B). The heatmap showed the overall distribution of DEGs (Figure 5C).

**FIGURE 6:**
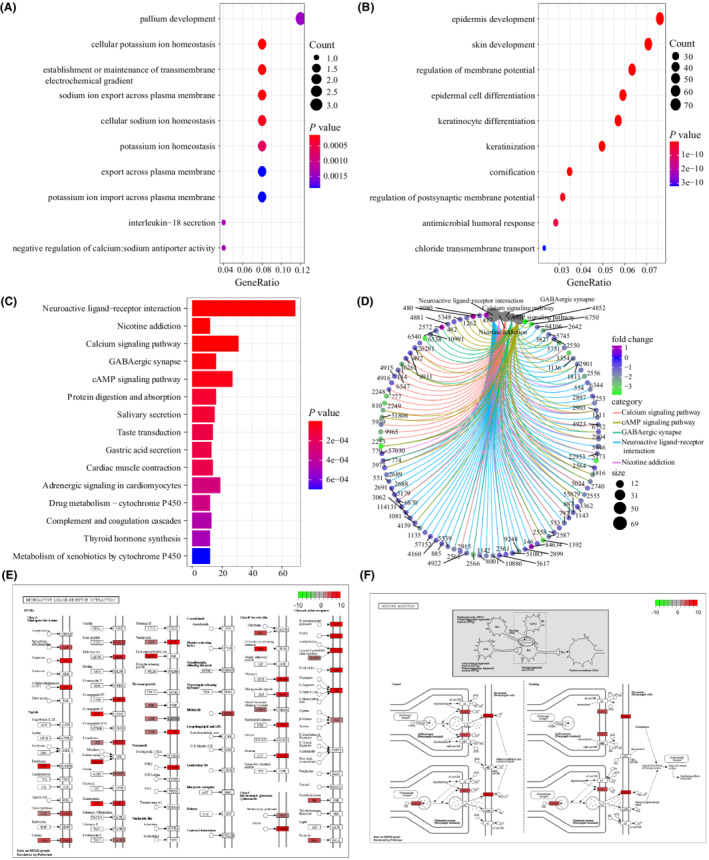
Functional enrichment analysis of DEGs based on *FAT2* mutation. (A) Gene Ontology (GO) analysis results of differentially expressed upregulated genes; (B) GO analysis results of differentially expressed downregulated genes; (C) Kyoto Encyclopedia of Genes and Genomes (KEGG) analysis indicated that these DEGs participated in neuroactive ligand‐receptor interaction, nicotine addiction, calcium signaling pathway, and biologically related signaling pathways such as GABAergic synapse. (D) KEGG pathway network diagram. The number represented the Entrez ID of the gene; (E, F) The factor with the highest degree of enrichment was neuroactive ligand‐receptor interactions and the nicotine addiction pathway.

GO analysis showed that those differentially upregulated genes were closely associated with biological processes such as cellular potassium ion homeostasis, establishment or maintenance of transmembrane electrochemical gradients, and sodium ion export across plasma membranes (Figure 6A). Differentially downregulated genes had a close correlation to biological processes such as epidermis development, skin development, and regulation of membrane potential (Figure 6B). The KEGG functional analysis suggested that DEGs mainly affected neuroactive ligand‐receptor interaction, nicotine addiction, calcium signaling pathway, GABAergic synapses, and cAMP signaling pathway (Figure 6C,D). The enrichment of neuroactive ligand‐receptor interaction and nicotine addiction are exhibited in Figure 6E,F.

**FIGURE 7:**
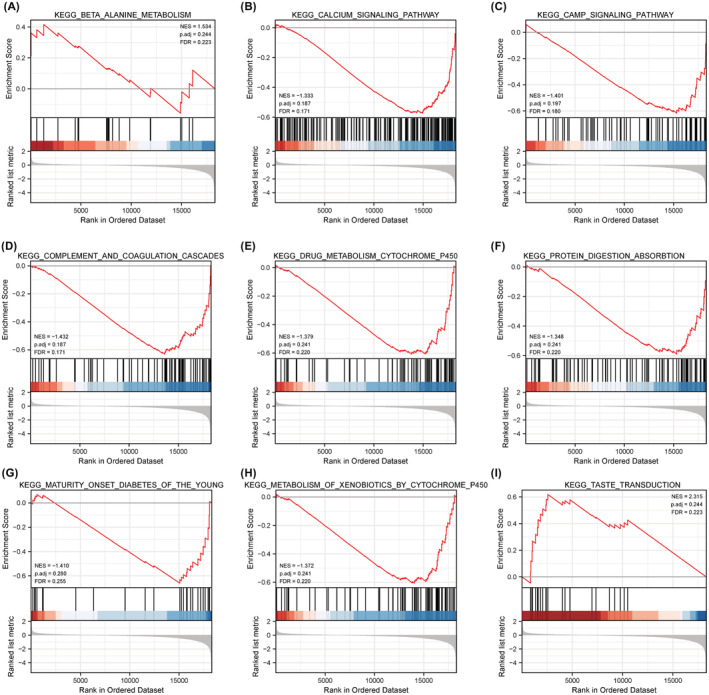
Gene set enrichment analysis (GSEA) of DEGs based on the TCGA‐UCEC dataset. GSEA results show that *FAT2* mutant tumor tissues are closely related to pathways such as Beta‐alanine metabolism, cAMP signaling pathway, calcium signaling pathway, and complement and coagulation cascades.

The GSEA results intimated that beta‐alanine metabolism, cAMP signaling pathway, calcium signaling pathway, and complement and coagulation cascades were enriched in *FAT2* mutant tissues significantly (Figure 7).

### 3.6 PPI network diagram and related regulation network's construction

**FIGURE 8:**
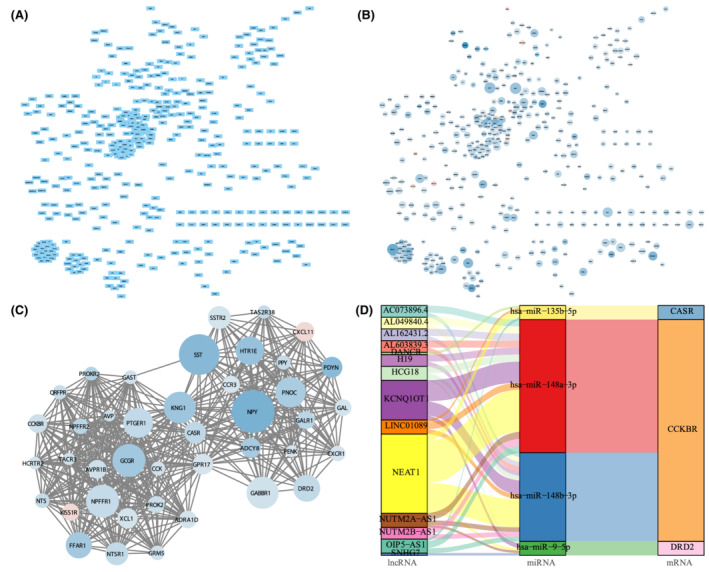
Protein–protein interaction (PPI) and construction of competing endogenous RNA (ceRNA) network. (A) STRING database analyzed the PPI network of DEGs; (B) Importing the analysis results into Cytoscape software. The red represented upregulated genes, and blue represented downregulated genes. The color depth was positively correlated with logFC, and the size of the circle was directly proportional to −log (*p* value); (C) Using the MCODE algorithm identified and extract local high‐density areas from the PPI network, where the size of the circle was proportional to −log (*p* value), and the color depth was proportional to logFC; (D) The Sankey diagram showed the construction of ceRNA interaction network based on hub genes.

The STRING database was employed to establish PPI network among DEGs (Figure 8A). Cytoscape further showed the interaction relationship among genes (Figure 8B). Hub genes, such as ADRA1D, KNG1, CASR, and GPR17, are shown in Figure 8C. Relied on the information about miRNA‐mRNA interaction and the hub genes obtained via the PPI network, we built a ceRNA network of miRNA‐mRNA‐lncRNA interactions and revealed a connection between lncRNA NEAT1, KCNQ1OT to miRNA has‐miR‐148a‐3p, has‐miarR‐148b‐3p and mRNA CCKBR (Figure 8D).

### 3.7 Association of FAT2 mutation with immune cell infiltrations and immunotherapy‐related target genes

**FIGURE 9:**
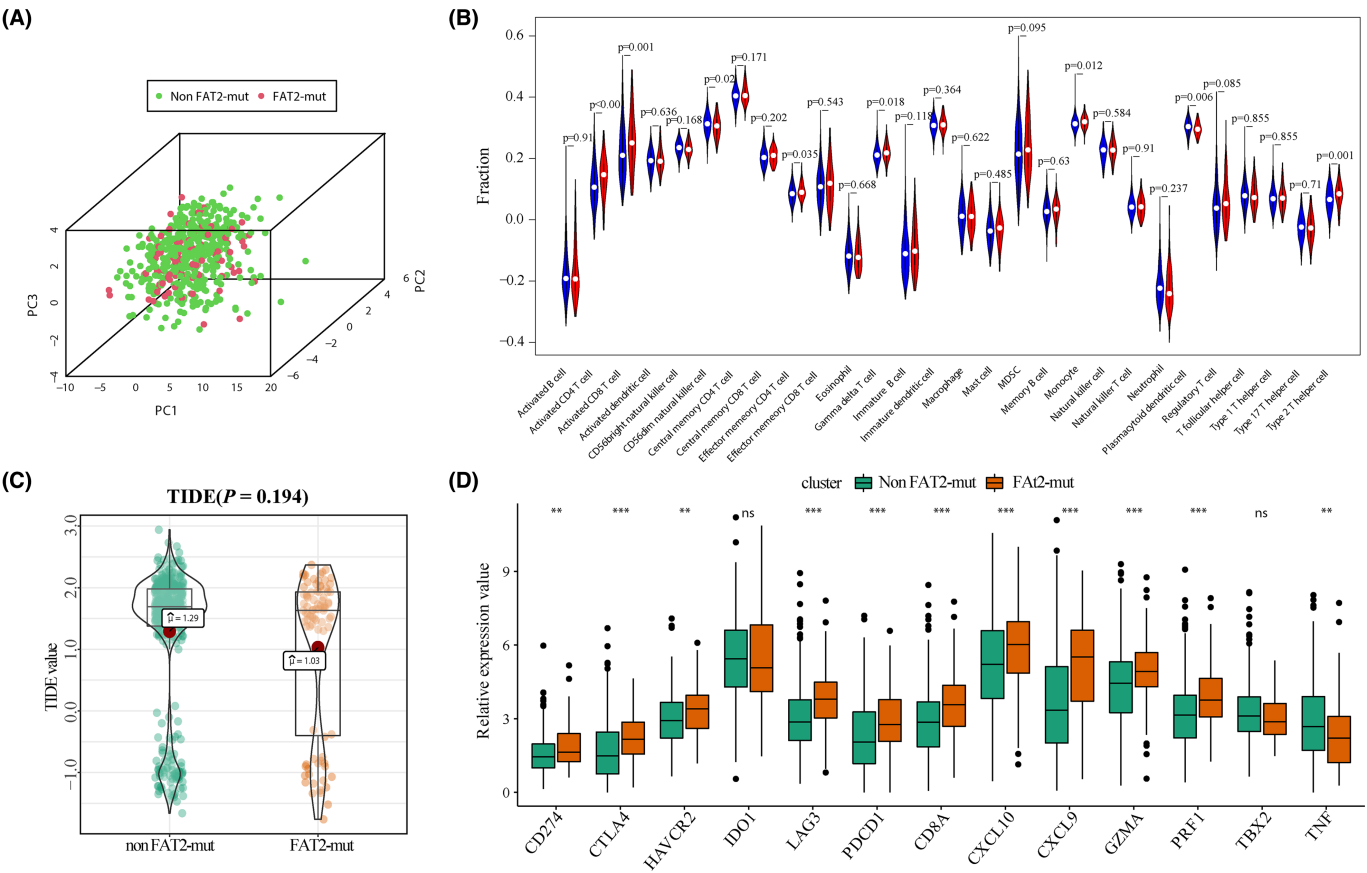
Effect of *FAT2* gene mutation in TCGA‐UCEC dataset on immunological characteristics. (A) According to principal component analysis, there was a certain difference in the overall immune cell infiltration level between the *FAT2* mutated group and the non‐*FAT2* mutated group; (B) The violin chart displayed the difference analysis of the levels of immune cell infiltration in these two groups, where red represented patients with *FAT2* mutations, and blue represented patients with non‐*FAT2* mutations; (C) There was no significant difference in the tracking of indels by decomposition value of immunotherapy‐related scores between patients with *FAT2* mutations and non‐*FAT2* mutations (*p* = 0.194); (D) Expression of multiple immunotherapy‐related target genes had a certain significant difference between patients with *FAT2* mutations and non‐*FAT2* mutations.

We explored the relationship between *FAT2* mutation and infiltration of TICs. The principal component analysis results showed that there were certain differences in infiltrated TICs between the *FAT2* mutations and non‐*FAT2* mutations groups (Figure 9A). In the TCGA‐UCEC dataset, the infiltration levels of activated CD4/CD8 T cells and plasmacytoid dendritic cells were significantly increased in the non‐*FAT2* mutation group, and Type 2 helper T cells were significantly decreased in the *FAT2* mutation group (Figure 9B). Although we saw differences in the level of immune cell infiltration with different *FAT2* mutation status in UCEC, the TIDE value was lower in the *FAT2* mutation group, indicating the *FAT2* mutation group showed higher immunotherapy response, but there is no significant difference between these groups (*p* = 0.194) (Figure 9C). Lots of immunotherapy‐related target genes were significantly upregulated in the *FAT2* mutation group, including *CTLA‐4, LAG3, PDCD1, CD8A, CXCL9/10, GZMA*, and *PFR1* (Figure 9D).

## 4 DISCUSSION

In the present study, 531 UCEC samples from TCGA database were utilized for analyzing the somatic mutation landscape. *FAT2* mutation showed a relatively high frequency in those TCGA samples, which was associated with better OS and DFS for 1, 3, and 5 years, and was an independent prognostic factor for patients with UCEC. We further identified that *FAT2* mutant UCEC had higher TMB and MSI scores. Several immunotherapy‐related target genes were significantly upregulated in the *FAT2* mutation group, such as *CTLA‐4, LAG3, PDCD1, CD8A, CXCL9/10, GZMA*, and *PFR1*. The GO analysis and GSEA results explored the potential mechanism of FAT2 mutation affecting tumorigenesis and progression of UCEC, which may give powerful clues for further research.

In clinical practice of UCEC treatment, risk stratification was built according to the criteria proposed by the ESMO‐ESGO‐ESTRO 2016 consensus, which separated four groups on the basis of clinicopathological features to recommend subsequent adjuvant therapy.^30^ Nevertheless, applying these assessment models alone lacks sufficient power to divide patients into high‐risk or low‐risk groups to docking subsequent strategy of adjuvant therapy.^31^, ^32^ There was growing evidence that combining clinicopathological features with molecular biomarkers to predict tumor behavior in patients could provide more accurate risk stratification and thereby improved patient outcomes.^33^, ^34^ The most commonly mutated genes that served as potential biomarkers in UCEC patients are *PTEN* and *TP53*.^35^ In this study, we found that OS and DFS were improved in patients with *FAT2* mutations, and *FAT2* gene mutation was an independent protective factor in patients with UCEC. Notably, *FAT2*‐mutated patients had fewer *TP53* mutations, which tend to be accompanied with higher grade UCEC and worse prognosis, which may be intrinsically closely related to our findings. In addition, in the study of chemotherapy drugs and small‐molecule targeted drugs, we found that patients with *FAT2* mutations have enhanced sensitivity to these drugs, indicating that postoperative adjuvant therapy may be more effective for this group of patients, thereby reducing the recurrence rate and improving OS. Our results were consistent with findings in other tumor studies investigating gastric cancer, LUAD, and NSCLC, for which patients with *FAT2* mutations had a better prognosis.^11^, ^12^, ^17^ Hence, we consider *FAT2* mutation status as a potential prognostic factor to guide the treatment of UCEC.

Although an increasing number of studies had indicated the important role of the *FAT2* gene on the survival and prognosis of patients with many types of tumors, there are few studies to investigate the specific mechanism underlying. In a previous study, *FAT2* was found to share the signaling pathway like Ena/VASP, which affects progression of gastric and pancreatic carcinoma.^10^ There were also studies showing that, as an atypical cadherin, *FAT2* could exert biological functions through the Hippo pathway, which is closely related to the maintenance of tissue homeostasis and to multiple processes including cancers.^36^, ^37^ In our study, patients with *FAT2* mutations were identified enriched in the cAMP and calcium signaling pathway, and cAMP and calcium signaling pathways participated in cell proliferation, migration, and metastasis of UCEC.^38^, ^39^ Moreover, we investigated the expression differences of various immune cells between *FAT2* mutation and wild‐type tumor tissues. We found higher expression of activated CD4/CD8 T cells and plasmacytoid dendritic cells in *FAT2* mutation patients. The tumor immune microenvironment (TIME) plays a pivotal role in tumorigenesis and progression of malignant tumor. Cytotoxic CD8 T cells exert potent anticancer immune responses by detecting intracellular antigens which were presented by major histocompatibility complex (MHC).^40^ By enhancing the killing effect of cytotoxic T lymphocytes (CTLs), CD4 T cells allow CTLs to overcome more obstacles and exert anticancer immune effects.^41^ Accordingly, changes in cell signaling pathways and TIME may be involved in the potential mechanism for the better prognosis of patients with *FAT2* mutations, but further experiments are needed to verify this hypothesis.

The treatment of recurrent and advanced UCEC is clinically challenging, and new therapies with innovative mechanisms have been explored to improve the prognosis of these patients. Among them, immunotherapy appears to be the most promising. Pembrolizumab, hindering PD‐1 binding to its ligands, was approved by the Food and Drug Administration to treat MMRd/MSI‐H and TMB‐H UCEC. MSI‐H/dMMR has been found in many different types of cancer including UCEC. MSI‐H/dMMR occurs when MMR proteins become dysfunctional for repairing DNA replication mistakes in microsatellites. TMB, usually measured by targeted genome sequencing or whole‐exome sequencing, has been applied to predict responsiveness of immunotherapy response in several cancer types.^42^ Previous studies have shown that higher TMB levels were associated with better effect of immunotherapy.^43^, ^44^ In our research, we found that UCEC patients with *FAT2* mutations had higher TMB and MSI, indicating that they might be more responsive to ICI therapy. In addition, we found that the *FAT2* gene affected immune targets, such as CTLA4. Taken together, we believe that the mutation status of *FAT2* may potentially be a marker for responsiveness of immunotherapy.

However, this study has several limitations. First, the findings were developed based on public datasets. Many of these data were missing, such as data on postoperative adjuvant therapy, which prevented us from performing further analyses, potentially biasing the results. At the same time, since the populations in the database are from developed countries, caution is required when applying the findings of this study to clinical scenarios in developing countries. Second, the lack of independent validation of clinical samples, especially for those receiving immunotherapy led to limited clinical value of this study. Finally, in‐depth biology experiment is needed to investigate the potential functions and molecular biology mechanism of FAT2 proteins in UCEC.

## 5 CONCLUSIONS

By comprehensively analyzing somatic mutation data of UCEC samples in the TCGA database, we deem *FAT2* mutation could serve as a prognostic indicator for UCEC. UCEC patients with *FAT2* mutations have higher MSI and TMB values and thus are more responsive to immunotherapy. Our findings give insights into the clinical implications of *FAT2* mutations in UCEC and lay the foundation for further research.

## AUTHOR CONTRIBUTIONS

Conception and design: Zhe Wang and Peilin Han. Acquisition of data: Zhe Wang and Linan Xing. Analysis and interpretation of data: Linan Xing and Yujie Huang. Writing the manuscript: Linan Xing, Zhe Wang, and Peilin Han. Study supervision: Peilin Han.

## FUNDING INFORMATION

This work was supported by grant from the National Natural Science Foundation of China (81902269).

## CONFLICT OF INTEREST

The authors declare that they have no competing interests.

## ETHICS APPROVAL

Not applicable.

## CONSENT FOR PUBLICATION

All authors agree for publication.

## Supporting information


Figure S1

Table S1

Table S2

Table S3

Table S4

## Data Availability

The data used and analyzed during this study are available from the corresponding author on request.
