# Supplementary figures and images for: FAT2 mutation is associated with better prognosis and responsiveness to immunotherapy in uterine corpus endometrial carcinoma
Source: Cancer Med. 2022 Aug 7;12(3):3797–811. doi: 10.1002/cam4.5119 (PMC9939103; doi:10.1002/cam4.5119)

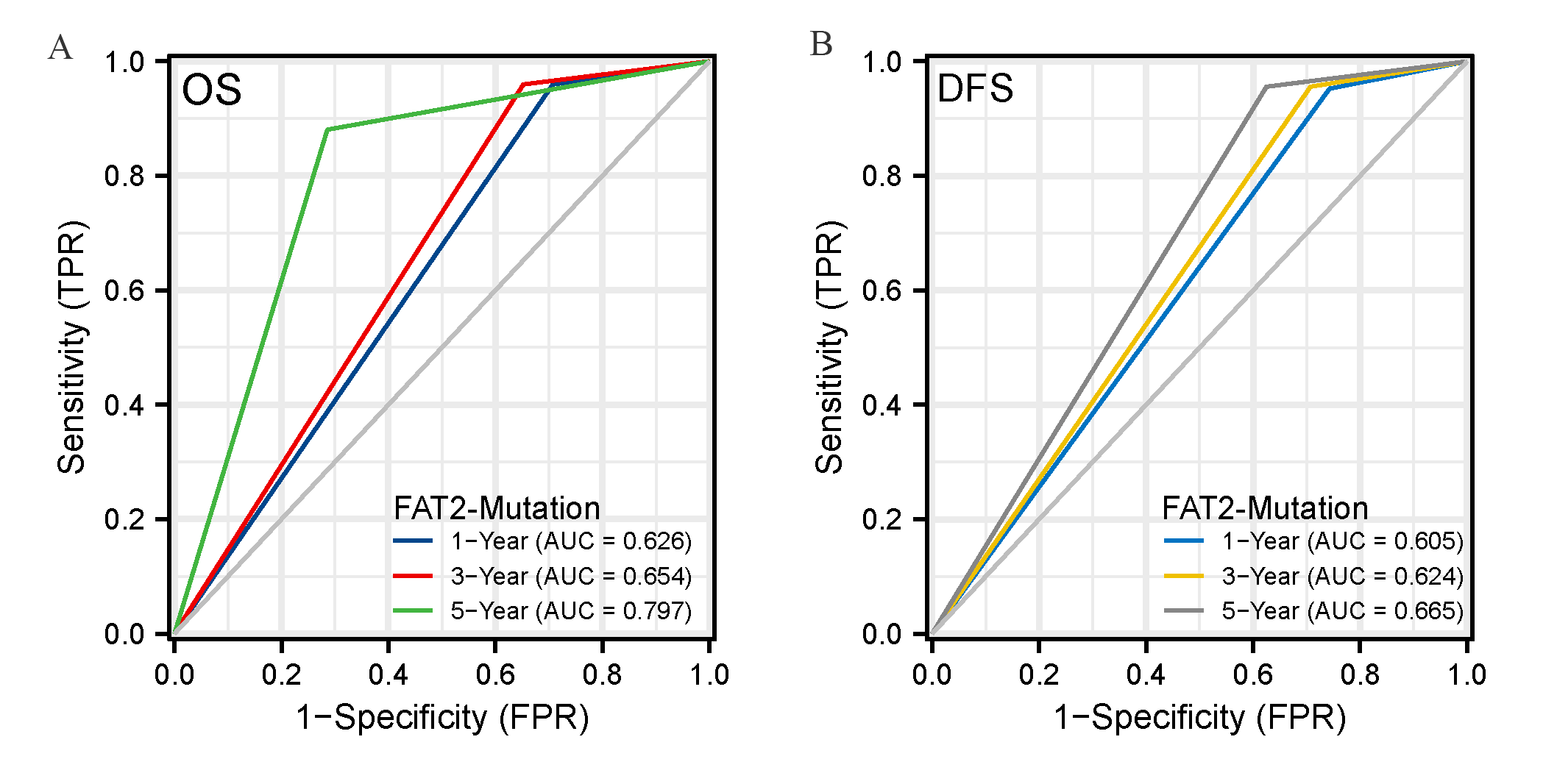

Supplement: Supplementary file 1 — Figure S1 [file CAM4-12-3797-s004.tif]
