# Supplementary material for: FAT2 mutation is associated with better prognosis and responsiveness to immunotherapy in uterine corpus endometrial carcinoma
Source: Cancer Med. 2022 Aug 7;12(3):3797–811. doi: 10.1002/cam4.5119 (PMC9939103; doi:10.1002/cam4.5119)
Supplement: Supplementary file 2 — Table S1 [file CAM4-12-3797-s005.docx]

TABLE S1. GO differential DEGs' analysis

| ONTOLOGY | ID | Description | Count | P value | Gene |
| --- | --- | --- | --- | --- | --- |
| Up-regulated DEGs | | | | | |
| BP | GO:0030007 | cellular potassium ion homeostasis | 2 | 0.000133 | ATP1A4/ATP1A2 |
| BP | GO:0010248 | establishment or maintenance of transmembrane electrochemical gradient | 2 | 0.000155 | ATP1A4/ATP1A2 |
| CC | GO:0005890 | sodium:potassium-exchanging ATPase complex | 2 | 0.000109 | ATP1A4/ATP1A2 |
| CC | GO:0090533 | cation-transporting ATPase complex | 2 | 0.000174 | ATP1A4/ATP1A2 |
| MF | GO:0005391 | sodium:potassium-exchanging ATPase activity | 2 | 0.000113 | ATP1A4/ATP1A2 |
| MF | GO:0008556 | potassium transmembrane transporter activity, phosphorylative mechanism | 2 | 0.000113 | ATP1A4/ATP1A2 |
| Down-regulated DEGs | | | | | |
| BP | GO:0008544 | epidermis development | 72 | 1.41E-17 | KRT31/KLK5/KRT83/WNT16/FOXN1/RPTN/BNC1/KRT85/POU2F3/TCHH/KRT81/SPINK9/KLK7/PKP1/CALML5/KRT33B/KRT36/DKK4/KRT38/KRTAP5-10/APCDD1/KRT34/PRR9/CRABP2/KRT84/FLG/WNT10A/REG3A/KRTDAP/KRT28/GDF3/KRTAP4-1/BMP4/KRT27/KRT72/KRT35/KRT76/KRTAP1-5/DSG3/KRT39/KLK12/ALX4/GAL/NTF4/KRTAP5-11/SPINK6/KRTAP5-7/REG3G/SERPINB13/KRTAP5-5/SPRR2G/DSG4/FLG2/KRT37/KRTAP11-1/FOXE1/KRTAP3-3/S100A7/KRTAP3-2/LCE5A/KRT71/SPRR2E/KRT74/SOX21/HOXC13/KRT77/KRT82/KRTAP3-1/KRT73/KRTAP10-12/SPRR4/KRTAP9-4 |
| BP | GO:0043588 | skin development | 67 | 4.12E-17 | KRT31/KLK5/KRT83/WNT16/FOXN1/RPTN/KRT85/TCHH/KRT81/SPINK9/PKP1/KRT33B/KRT36/DKK4/COMP/KRT38/KRTAP5-10/APCDD1/KRT34/PRR9/ALOX12B/KRT84/FLG/WNT10A/REG3A/KRT28/KRTAP4-1/KRT27/RYR1/KRT72/KRT35/KRT76/KRTAP1-5/DSG3/KRT39/KLK12/ALX4/GAL/KRTAP5-11/SPINK6/KRTAP5-7/REG3G/SERPINB13/KRTAP5-5/SPRR2G/DSG4/FLG2/KRT37/KRTAP11-1/FOXE1/KRTAP3-3/S100A7/KRTAP3-2/LCE5A/KRT71/SPRR2E/KRT74/SOX21/HOXC13/KRT77/KRT82/KRTAP3-1/KRT73/KRTAP10-12/SPRR4/ASCL4/KRTAP9-4 |
| CC | GO:1902495 | transmembrane transporter complex | 59 | 6.67E-18 | CACNA1S/ANO2/KCNIP1/TTYH1/CHRNA3/KCNJ4/KCNQ2/GRIK5/CACNA2D3/GABRA3/KCNK2/GRIK1/CACNG6/ATP1B2/GRIN2A/KCNE5/SCN11A/SCN5A/SCN2B/GRIN2B/CACNA1E/CLCNKB/SNAP25/GABRE/KCNB1/SCN4A/FXYD1/LRRC38/CATSPERD/HTR3B/GABRA2/KCNQ5/CHRNB4/LRRC55/GABRQ/BEST3/SCN2A/RYR1/ATP12A/GABRA5/KCNA2/CACNA1B/KCNB2/GRIK3/DPP6/NLGN1/LRRC52/CFTR/CACNA1I/GABRB2/HTR3A/CHRNB3/GLRA3/GABRG2/GABRG1/KCNC2/CHRNA2/OLFM3/CACNG3 |
| CC | GO:0034702 | ion channel complex | 56 | 1.72E-17 | CACNA1S/ANO2/KCNIP1/TTYH1/CHRNA3/KCNJ4/KCNQ2/GRIK5/CACNA2D3/GABRA3/KCNK2/GRIK1/CACNG6/GRIN2A/KCNE5/SCN11A/SCN5A/SCN2B/GRIN2B/CACNA1E/CLCNKB/SNAP25/GABRE/KCNB1/SCN4A/LRRC38/CATSPERD/HTR3B/GABRA2/KCNQ5/CHRNB4/LRRC55/GABRQ/BEST3/SCN2A/RYR1/GABRA5/KCNA2/CACNA1B/KCNB2/GRIK3/DPP6/NLGN1/LRRC52/CFTR/CACNA1I/GABRB2/HTR3A/CHRNB3/GLRA3/GABRG2/GABRG1/KCNC2/CHRNA2/OLFM3/CACNG3 |
| MF | GO:0022836 | gated channel activity | 66 | 1.08E-20 | CACNA1S/ANO2/KCNT1/KCNIP1/TTYH1/CHRNA3/KCNJ4/TRPM5/KCNK9/KCNQ2/JPH3/GRIK5/CACNA2D3/GABRA3/KCNJ15/KCNK2/GRIK1/CACNG6/GRIN2A/KCNE5/SCN11A/SCN5A/SCN2B/GRIN2B/CACNA1E/KCNT2/P2RX2/CLCNKB/SNAP25/GABRE/KCNB1/SCN4A/LRRC38/P2RX3/CNGA4/HTR3B/GABRA2/KCNQ5/CHRNB4/LRRC55/GABRQ/SCN2A/RYR1/GABRA5/KCNA2/CACNA1B/CLCA2/KCNB2/GRIK3/SLC17A7/KCNJ12/LRRC52/CFTR/CACNA1I/GABRB2/HTR3A/CHRNB3/GLRA3/GABRG2/HTR3C/GABRG1/KCNC2/CHRNA2/SLC17A3/CACNG3/CLCA1 |
| MF | GO:0022803 | passive transmembrane transporter activity | 77 | 2.79E-19 | CACNA1S/ANO2/KCNT1/KCNIP1/TTYH1/CHRNA3/KCNJ4/TRPM5/KCNK9/GPM6A/KCNQ2/JPH3/GRIK5/CACNA2D3/GABRA3/KCNJ15/KCNK2/GRIK1/CACNG6/GRIN2A/KCNE5/SCN11A/SCN5A/SLC5A8/SCN2B/GRIN2B/CACNA1E/KCNT2/P2RX2/SLC14A2/CLCNKB/SNAP25/GABRE/KCNB1/SCN4A/FXYD1/LRRC38/P2RX3/CNGA4/HTR3B/GABRA2/KCNQ5/CHRNB4/LRRC55/GABRQ/BEST3/SCN2A/RYR1/GABRA5/KCNA2/CACNA1B/CLCA2/KCNB2/GRIK3/SLC17A7/GJA8/KCNJ12/LRRC52/CFTR/CACNA1I/GABRB2/HTR3A/CHRNB3/GLRA3/GABRG2/TRPV5/HTR3C/GABRG1/KCNC2/CHRNA2/BSND/SLC17A3/AQP10/RHAG/CACNG3/AQP12A/CLCA1 |

BP: Biological Process

CC: Cellular Component

MF: Molecular Function

ID: Entrez Id
