# Supplementary material for: FAT2 mutation is associated with better prognosis and responsiveness to immunotherapy in uterine corpus endometrial carcinoma
Source: Cancer Med. 2022 Aug 7;12(3):3797–811. doi: 10.1002/cam4.5119 (PMC9939103; doi:10.1002/cam4.5119)
Supplement: Supplementary file 3 — Table S2 [file CAM4-12-3797-s001.docx]

Table S2. KEGG differential DEGs'analysis.

| ID | Description | Coun  t | P value | Gene ID |
| --- | --- | --- | --- | --- |
| hsa04080 | Neuroactive ligand-receptor interaction | 71 | 6.95E-24 | NPY/SST/NPFFR1/GCGR/PTH1R/GABBR1/KNG1/PTGER1/HTR1E/CHRNA3/DRD2/GRIK5/GABRA3/AVPR2/SCTR/GRIK1/TSHR/GRIN2A/GPHA2/CTSG/NTSR1/SSTR2/GRIN2B/PRSS3/P2RX2/PDYN/GABRE/DRD5/GLP1R/P2RX3/GABRA2/HTR6/CHRNB4/GABRQ/CCKBR/CALCB/AVPR1B/GABRA5/GALR1/CRH/ADRA1D/KISS1R/GRIK3/GPR50/GAL/GABRB2/PRL/NPFFR2/CHRNB3/GLRA3/GABRG2/NTS/GRM5/GABRG1/PPY/CCK/MC4R/GPHB5/SLURP1/CHRNA2/MC3R/CGA/UCN3/HCRTR2/GHRH/TACR3/PENK/GH1/AVP/GH2/LHCGR |
| hsa05033 | Nicotine addiction | 12 | 5.75E-07 | GABRA3/GRIN2A/GRIN2B/GABRE/GABRA2/GABRQ/GABRA5/CACNA1B/SLC17A7/GABRB2/GABRG2/GABRG1 |
| hsa04020 | Calcium signaling pathway | 31 | 2.94E-06 | CACNA1S/FGF8/PTGER1/FGF19/RET/CALML5/GRIN2A/NTSR1/CALML3/FGF4/FGF3/CACNA1E/NTRK3/P2RX2/NTRK2/DRD5/SLC8A3/ADCY8/P2RX3/HTR6/CCKBR/AVPR1B/RYR1/ADRA1D/CACNA1B/CACNA1I/ATP2B3/GRM5/FGF20/TACR3/LHCGR |
| hsa04024 | cAMP signaling pathway | 29 | 4.50E-06 | NPY/SST/CACNA1S/GABBR1/HTR1E/DRD2/NPR1/CALML5/TSHR/ATP1B2/GRIN2A/GPHA2/SSTR2/CALML3/GRIN2B/DRD5/FXYD1/GLP1R/ADCY8/CNGA4/HTR6/ATP1A4/CRH/CFTR/ATP2B3/ATP1A2/GPHB5/CGA/LHCGR |
| hsa04727 | GABAergic synapse | 16 | 1.36E-05 | SLC6A11/CACNA1S/GABBR1/SLC38A3/GABRA3/GABRE/ADCY8/GABRA2/SLC6A13/GABRQ/GABRA5/CACNA1B/GABRB2/GABRG2/GABRG1/GAD2 |
| hsa04974 | Protein digestion and absorption | 16 | 8.75E-05 | COL4A3/PGA5/COL9A3/COL4A4/CPB1/ATP1B2/COL22A1/PRSS3/SLC8A3/MEP1A/COL9A1/ATP1A4/ATP1A2/SLC6A19/SLC36A2/SLC36A3 |
| hsa04970 | Salivary secretion | 15 | 9.35E-05 | CST4/CST1/DMBT1/CALML5/ATP1B2/LPO/CALML3/CST2/ADCY8/ATP1A4/ADRA1D/ATP2B3/ATP1A2/CST5/STATH |
| hsa04742 | Taste transduction | 14 | 0.0001439 | GABBR1/HTR1E/TRPM5/GABRA3/P2RX2/ADCY8/P2RX3/HTR3B/GABRA2/SCN2A/GABRA5/HTR3A/HTR3C/TAS2R38 |
| hsa04971 | Gastric acid secretion | 13 | 0.0001479 | SST/CALML5/KCNJ15/KCNK2/ATP1B2/SSTR2/CALML3/ADCY8/CCKBR/ATP1A4/CFTR/ATP1A2/GAST |
