# Supplementary material for: FAT2 mutation is associated with better prognosis and responsiveness to immunotherapy in uterine corpus endometrial carcinoma
Source: Cancer Med. 2022 Aug 7;12(3):3797–811. doi: 10.1002/cam4.5119 (PMC9939103; doi:10.1002/cam4.5119)
Supplement: Supplementary file 4 — Table S3 [file CAM4-12-3797-s002.docx]

Table S3. Gene set enrichment analysis

| Description | S  i  ze | Enrichment Score | NES | P value | Leading_edge |
| --- | --- | --- | --- | --- | --- |
| KEGG_BETA_ALANINE_METABOLISM | 22 | 0.41657588 | 1.53362158 | 0.02 | tags=23%, list=7%, signal=21% |
| KEGG_CAMP_SIGNALING_PATHWAY | 177 | -0.57320456 | -1.33325528 | 0.003996 | tags=36%, list=19%, signal=29% |
| KEGG_CALCIUM_SIGNALING_PATHWAY | 73 | -0.61688597 | -1.40131991 | 0.00501505 | tags=36%, list=16%, signal=30% |
| KEGG_COMPLEMENT_AND_COAGULATION_CASCADES | 69 | -0.63170451 | -1.43209358 | 0.00401204 | tags=58%, list=26%, signal=43% |
| KEGG_DRUG_METABOLISM_CYTOCHROME_P450 | 68 | -0.60767722 | -1.3788239 | 0.00802407 | tags=56%, list=25%, signal=42% |
| KEGG_PROTEIN_DIGESTION_ABSORBTION | 83 | -0.59042176 | -1.34821235 | 0.01201201 | tags=31%, list=16%, signal=26% |
| KEGG_MATURITY_ONSET_DIABETES_OF_THE_YOUNG | 25 | -0.66106898 | -1.40998188 | 0.02579979 | tags=56%, list=18%, signal=46% |
| KEGG_METABOLISM_OF_XENOBIOTICS_BY_CYTOCHROME_P450 | 66 | -0.60652399 | -1.37169291 | 0.01003009 | tags=55%, list=23%, signal=42% |
| KEGG_TASTE_TRANSDUCTION | 23 | 0.61842249 | 2.31477694 | 0.01960784 | tags=52%, list=14%, signal=45% |
