# Supplementary material for: FAT2 mutation is associated with better prognosis and responsiveness to immunotherapy in uterine corpus endometrial carcinoma
Source: Cancer Med. 2022 Aug 7;12(3):3797–811. doi: 10.1002/cam4.5119 (PMC9939103; doi:10.1002/cam4.5119)
Supplement: Supplementary file 5 — Table S4 [file CAM4-12-3797-s003.docx]

Table S4. Univariate and multivariate Cox analysis risk scores of UCEC patients on TCGA data

|  | Univariate Cox analysis | | | | Multivariate Cox analysis | | | |
| --- | --- | --- | --- | --- | --- | --- | --- | --- |
|  | HR | HR.95L | HR.95H | *P* value | HR | HR.95L | HR.95H | *P* value |
| Age (≥60 vs. <60) | 2.05 | 1.22 | 3.44 | 0.006833 | 1.15 | 0.66 | 1.99 | 0.626354 |
| pStage (Stage II vs. Stage I) | 1.92 | 0.87 | 4.20 | 0.104697 | 1.54 | 0.70 | 3.39 | 0.28472 |
| pStage (Stage III vs. Stage I) | 3.71 | 2.25 | 6.12 | 2.94E-07 | 3.40 | 2.03 | 5.70 | 3.32E-06 |
| pStage (Stage IV vs. Stage I) | 9.26 | 5.05 | 16.96 | 5.71E-13 | 5.87 | 3.15 | 10.96 | 2.65E-08 |
| Grade (G2 vs. G1) | 6.78 | 1.53 | 30.07 | 0.011742 | 5.67 | 1.27 | 25.24 | 0.022859 |
| Grade (G3 vs. G1) | 13.64 | 3.34 | 55.61 | 0.000269 | 9.69 | 2.32 | 40.47 | 0.00184 |
| FAT2 Mut (Mut vs. Non-mut) | 0.24 | 0.10 | 0.60 | 0.002266 | 0.18 | 0.07 | 0.48 | 0.00061 |

HR: hazard ratio; Mut: mutant.
